# Vitamin B_12_ deficiency in the brain leads to DNA hypomethylation in the TCblR/CD320 knockout mouse

**DOI:** 10.1186/1743-7075-9-41

**Published:** 2012-05-18

**Authors:** Sílvia Fernàndez-Roig, Shao-Chiang Lai, Michelle M Murphy, Joan Fernandez-Ballart, Edward V Quadros

**Affiliations:** 1 Area of Preventive Medicine and Public Health, Faculty of Medicine and Health Sciences, Universitat Rovira i Virgili (URV), IISPV, Reus, Spain; 2 CIBER Fisiopatología de la Obesidad y Nutrición (CB06/03) Instituto Carlos III, Madrid, 28029, Spain; 3 Departments of Medicine and Cell Biology, SUNY Downstate Medical Center, Brooklyn, NY, USA; 4 Department of Medicine, SUNY Downstate Medical Center, Brooklyn, NY, USA

**Keywords:** Vitamin B_12_ deficiency, Brain, Global DNA methylation, Transcobalamin receptor knock out mice

## Abstract

**Background:**

DNA methylation is an epigenetic phenomenon that can modulate gene function by up or downregulation of gene expression. Vitamin B_12_ and folate pathways are involved in the production of S-Adenosylmethionine, the universal methyl donor.

**Findings:**

Brain vitamin B_12_ concentration and global DNA methylation was determined in transcobalamin receptor (TCblR/*CD320*) knock out (KO) (n = 4) and control mice (n = 4) at 20–24 weeks of age. Median [IQR] brain vitamin B_12_ concentrations (pg/mg) in TCblR/*CD320* KO mice compared with control mice was 8.59 [0.52] vs 112.42 [33.12]; p < 0.05. Global DNA methylation levels in brain genomic DNA were lower in TCblR/*CD320* KO compared with control mice (Median [IQR]: 0.31[0.16] % vs 0.55[0.15] %; p < 0.05.).

**Conclusions:**

In TCblR/*CD320* KO mice, brain vitamin B_12_ drops precipitously by as much as 90% during a 20 week period. This decrease is associated with a 40% decrease in global DNA methylation in the brain. Future research will reveal whether the disruption in gene expression profiles due to changes in DNA hypomethylation contribute to central nervous system pathologies that are frequently seen in vitamin B_12_ deficiency.

## Findings

### Background

Vitamin B_12_ and folate participate in a number of biochemical reactions that affect methyl group production and transfer. Vitamin B_12_ is a cofactor for methionine synthase (MS) that catalyses the conversion of homocysteine to methionine. Methionine is then converted to S-adenosylmethionine (SAM), a universal methyl group donor [1] for methylation of DNA and RNA. The transfer of a methyl group to the 5-carbon cytosine residue by SAM is catalysed by DNA methyltransferase enzymes [2].

DNA methylation is a fundamental mechanism for the epigenetic control of gene expression and the maintenance of genomic integrity [3]. DNA hypermethylation can lead to transcriptional silencing by blocking transcription factors [4] and DNA hypomethylation to genomic instability and the expression of genes that would be otherwise suppressed [5]. DNA methylation plays a critical role in the generation of oncogenic point mutations, silencing of tumor suppressor genes and in the activation of the cancer process [6].

Low vitamin B_12_ status results in reduced MS activity, impaired remethylation of homocysteine to methionine and subsequent SAM production. Consequently, methylation reactions are impaired and DNA methylation capacity is reduced [7]. Severe vitamin B_12_ deficiency leads to neurological manifestations such as myelopathy, neuropathy, dementia and neuropsychiatric abnormalities [8] and to demyelination in the central nervous system [9]. DNA hypomethylation could be a link between vitamin B_12_ deficiency and neurological pathology. Although the association was not maintained in multivariate analysis, preliminary analysis showed that low tissue B_12_ concentrations were associated with DNA hypomethylation in human squamous cell lung cancer [10]. B_12_ deficient diet has decreased DNA methylation and increased uracil incorporation in rats [11]. DNA hypomethylation has been reported in fetal brains with neural tube defects [12]. The transcobalamin receptor (TCblR)/*CD320* knock out (KO) mouse provides a model to investigate the relationship between severe B_12_ deficiency and DNA methylation status.

### Hypothesis

Considering the role of vitamin B_12_ in recycling of folate and *de novo* methionine production, our hypothesis is that vitamin B_12_ deficiency in the brain causes global DNA hypomethylation in the TCblR/*CD320* mouse.

## Methods

### Animals

The TCblR KO mouse ES cells (+/−) generated by gene trap technology at the Sanger Institute were obtained from the Mouse Genome Center, UC Davis, CA. The *CD320* gene KO was produced in the C57Bl/6 N strain using heterozygous ES cells. The KO mice were produced in the laboratory of R Finnell, Texas Institute of Genomic Medicine, Houston, TX. The null TCblR/*CD320* KO mouse was generated by cross breeding. The production of CD320 KO mice and breeding was done as per NIH guidelines and the protocol was approved by the AALAC accredited institutional animal care and use committee. The mice were bred and fed on standard chow at SUNY Downstate Medical Center (Brooklyn, New York) for the study. The initial genotyping was done by PCR using genomic DNA extracted from mouse tail. In this study, four control and four TCblR/*CD320* null mice were analyzed. The animals were euthanized by CO_2_ inhalation before being sacrificed at age 20–24 weeks. The whole brain was removed and quick-frozen in liquid nitrogen. After dissection, the brain tissue was stored at −80°C.

### Preparation of mouse tissue samples for total vitamin B_12_ assay

Brain tissue was homogenized in two volumes of distilled water based on wet weight of tissue. 100 μl of the homogenized tissue was mixed with nine volumes of vitamin B_12_ extraction buffer (one volume of 0.06 M citrate-phosphate buffer, pH 2.6, one volume of 0.1 M Na_2_HPO_4_, pH 7.4 and one volume of KCN, 100 μg/ml; final pH of extraction buffer is 4.5); in a tightly capped glass tube and placed in a boiling water bath for 30 minutes to extract vitamin B_12_. The sample was briefly centrifuged after being cooled to room temperature and transferred to a 1.5 ml microcentrifuge tube. The precipitated proteins were pelleted by spinning at 14,000 rpm for 15 minutes and the clear extract was collected and neutralized by mixing one-third of a volume of 0.2 M Na_2_HPO_4_, pH 9.0. Total vitamin B_12_ was assayed as previously described [13].

### Determination of global DNA methylation in the brain

Genomic DNA from 25 mg of brain tissue was purified with the *DNeasy blood & tissue kit* (Qiagen, Inc). DNA concentration and purity was determined by comparing the ratio of optical density measurements at 260 and 280 nm. Global DNA methylation was determined using the Methyl Flash Methylated DNA Quantification Kit (Colorimetric) (Epigentek Group Inc., New York, NY, USA) [14]. The kit measures the methyl-cytosine content as a percentage of total cytosine content. Using a DNA concentration of 20 μg/mL, the purified DNA was added to ELISA plate. The methylated fraction of DNA is quantified using 5-methylcytosine specific antibodies. The amount of methylated DNA was proportional to the OD intensity in an ELISA plate reader at 450 nm. DNA methylation was calculated using the formula: [(sample OD–M3OD)/S]/[((M4OD– M3OD)x2)/P]x100; where **OD** is optical density; **M3** is the negative control, an unmethylated polynucleotide containing 50% of cytosine; **S** is the amount of input sample DNA in ng; **M4** is the positive control, a methylated polynucleotide containing 50% of 5-methylcystosine; **P** is the amount of input positive control in ng. The amount of methylated DNA was expressed as percentage of total DNA.

### Statistical analysis

Vitamin B_12_ concentration and global DNA methylation are reported as median [IQR]. Both determinations were compared between control and TCblR/*CD320* KO mice groups with the non parametric one-tailed Mann Whitney U test*.* A p-value <0.05 was considered significant. The data were analyzed using SPSS *(Statistical Package for Social Sciences, Chicago, IL, USA)* version 17.0.

## Results and discussion

**Figure 1:**
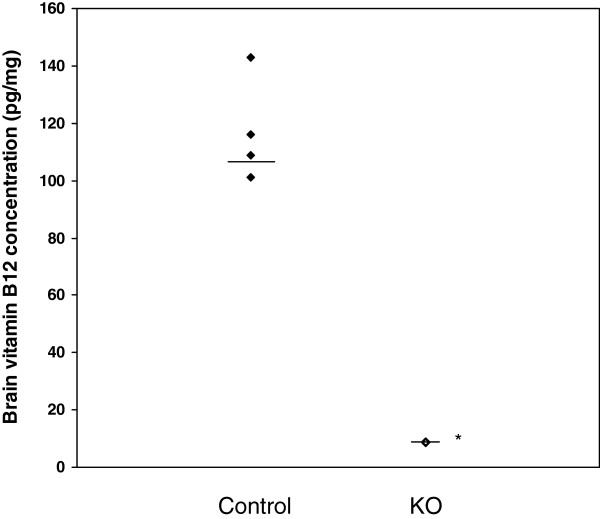
**Vitamin B**_**12**_**concentration in control and TCblR/*CD320*KO mouse brain.** Lines in graph represent the medians. *Mann Whitney U test, compared to control mice, p < 0.05, (n = 4).

**Figure 2:**
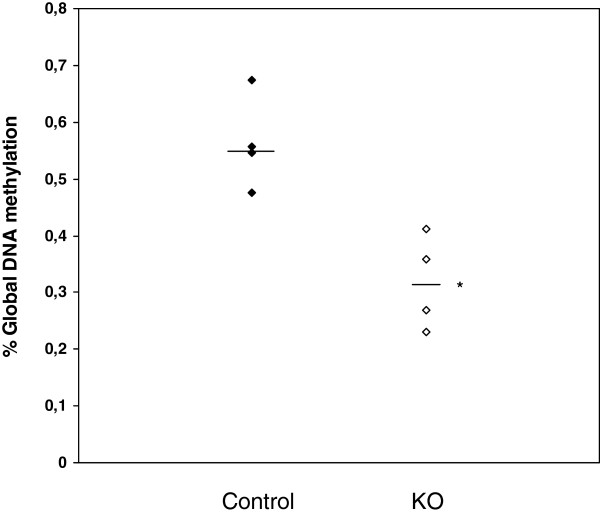
**Global DNA methylation in control and TCblR/*CD320*KO mouse brain.** Lines in graph represent the medians. * Mann Whitney U test, compared to control mice, p < 0.05, (n = 4).

Median [IQR] brain vitamin B_12_ concentrations in TCblR/*CD320* KO mice were lower than in control mice: 8.6 [0.52] pg/mg vs 112.4 [33.12] pg/mg; p < 0.05 (Figure 1). Brain vitamin B_12_ concentration was 92% lower in TCblR/*CD320* KO mouse brains than in controls. Median [IQR] global genomic DNA methylation levels were significantly lower in TCblR/*CD320* KO (0.31 [0.16] %) than control mice brains (0.55 [0.15] %) (p < 0.05) (Figure 2). Global DNA methylation was 44% lower in TCblR/*CD320* KO brain tissue than in control.

TCblR has an essential role in ensuring vitamin B_12_ bioavailability. Transcobalamin, the plasma vitamin B_12_ transporter, saturated with vitamin B_12_ binds to the TCblR, and is internalized by endocytosis [15]. The TCblR/*CD320* KO mice did not express functional TCblR, so cellular vitamin B_12_ uptake by this route was not possible. As a result, vitamin B_12_ depletion occurred in TCblR/*CD320* KO mouse brain but not in control brain that had normal TCblR expression. However, this gene knockout is not embryonic lethal and so far it appears that the mice appear to breed normally.

*Choi, et al*, demonstrated that vitamin B_12_ depletion in rats, following ten weeks on a vitamin B_12_-deficient diet, induced a 35% reduction in genomic DNA methylation of rat colonic epithelium compared with control rats [11]. The results from our study support this observation. We found that in the TCblR/*CD320* KO mice, with depleted vitamin B_12_ in the brain, global DNA methylation was 44% lower than in control vitamin B_12_ replete mice.

Methylcobalamin is a cofactor for MS, responsible for methyl transfer from homocysteine to methionine [16]. This metabolic pathway is impaired when vitamin B_12_ supply is low. As a result, SAH accumulates and this affects the ability of DNA methyltransferase enzymes to methylate cytosine residues and DNA hypomethylation occurs [17].

Vitamin B_12_ deficiency leads to defective myelin formation [9] and epigenetic factors, such as histone modifications, are involved in this process [18]. The change in DNA methylation pattern observed in the TCblR/*CD320* KO mouse brain might contribute to the demyelination process that occurs in advanced vitamin B_12_ deficiency. To confirm this hypothesis, the methylation pattern of genes involved in the myelination process and its association with changes in their gene expression should be investigated.

This preliminary study provides evidence of severe impairment in DNA methylation capacity in the brain of TCblR/*CD320* KO mice.

## Abbreviations

MS, Methionine synthase; SAM, S-adenosylmethionine; TCblR, Transcobalamin receptor; KO, Knock out; CNS, Central nervous system.

## Competing interests

EVQ is an inventor in a patent (WO2007/117657 A2) filed by the Research Foundation of SUNY. Other authors have no conflicts of interest.

## Author’s contributions

EQ: Principal investigator of the study and responsible for generating the hypothesis., EQ, SFR and SCL: Participated in the design and coordination of the study., SFR, SCL, EQ, JDFB, MM: Responsible for writing and statistical analysis., EQ, SCL, SFR, JDFB, MM: were involved in the analysis of the data. All authors read and approved the final version of the manuscript.

## Availability of supporting data

NA
